# Non-technical skills needed by medical disaster responders– a scoping review

**DOI:** 10.1186/s13049-024-01197-y

**Published:** 2024-04-02

**Authors:** Anja Westman, Lisa Kurland, Karin Hugelius

**Affiliations:** 1 Faculty of Medicine and Health, Örebro University, Örebro, Sweden; 2 Department of Emergency Care, Örebro University Hospital, Örebro, Sweden

**Keywords:** Disaster, Disaster responders, Non-technical skills, Competence

## Abstract

**Background:**

There is no universal agreement on what competence in disaster medicine is, nor what competences and personal attributes add value for disaster responders. Some studies suggest that disaster responders need not only technical skills but also non-technical skills. Consensus of which non-technical skills are needed and how training for these can be provided is lacking, and little is known about how to apply knowledge of non-technical skills in the recruitment of disaster responders. Therefore, this scoping review aimed to identify the non-technical skills required for the disaster medicine response.

**Method:**

A scooping review using the Arksey & O´Malley framework was performed. Structured searches in the databases PuBMed, CINAHL Full Plus, Web of Science, PsycInfo and Scopus was conducted. Thereafter, data were structured and analyzed.

**Results:**

From an initial search result of 6447 articles, 34 articles were included in the study. These covered both quantitative and qualitative studies and different contexts, including real events and training. The most often studied real event were responses following earthquakes. Four non-technical skills stood out as most frequently mentioned: communication skills; situational awareness; knowledge of human resources and organization and coordination skills; decision-making, critical-thinking and problem-solving skills. The review also showed a significant lack of uniform use of terms like skills or competence in the reviewed articles.

**Conclusion:**

Non-technical skills are skills that disaster responders need. Which non-technical skills are most needed, how to train and measure non-technical skills, and how to implement non-technical skills in disaster medicine need further studies.

**Supplementary Information:**

The online version contains supplementary material available at 10.1186/s13049-024-01197-y.

## Background

A frequently used definition of disasters is that they are “serious disruptions to the functioning of a community that exceed its capacity to cope using its own resources. Disasters can be caused by natural, man-made and technological hazards, as well as various factors that influence the exposure and vulnerability of a community” [1] Disaster strikes worldwide and recurs [2]. In 2022, 387 natural hazards and disasters worldwide were recorded and the increasing occurrence of disasters emphasizes the need to further address disaster preparedness [2]. 

Disaster response includes all actions from the first acute phase to operations with a longer timeline. Disaster response teams can therefore vary and be composed differently.

An important aspect of disaster response is to make sure competent disaster responders and disaster teams are available for disaster relief, both in the acute phase and for a prolonged period, depending on the specific task at hand. However, standardization with respect to disaster responders’ necessary competence is lacking [3]. 

Competence can be described as the ability to do something well and perform in relation to the task. The relationship between competence and skills can be described as competence being the combined result of skills, knowledge and abilities [4]. Skills may be technical (TS) and thereby task specific, such as knowing how to perform a measurable task (e.g. performing a specific surgical operation, intubating a patient, or suturing wounds, or non-technical skills (NTS), defined as “a constellation of cognitive and social skills, exhibited by individuals and teams, needed to reduce error and improve human performance in complex systems” [5, 6]. The concept of NTS stems from the development of crew resource management (CRM) and adapted for medicine from the 1990s onwards [5]. NTS have been defined as a set of seven skills (situation awareness; decision-making; communication, teamwork, leadership, managing stress and coping with fatigue) [7]. However, some sources describe six topics (performance-shaping factors; planning, preparation and prioritizing; situation awareness and perception of risk; decision-making; communication and teamwork and leadership) [5], while sometimes a wider thematic approach is adopted (personal resource skills, interpersonal skills, and cognitive skills) [8]. 

Some areas of medicine have accepted NTS and incorporated them into their curriculum (e.g. within anesthesia and critical care), but their value has not yet been widely accepted nor are they used in the settings of disaster response or disaster medicine competence.

NTS are both closely connected to pure (TS) but also intertwined and in a way inseparable. Both TS and NTS can be trained or learned. However, NTS have been suggested to play a central role in the performance of crises response teams in general [9]. Nevertheless, most studies on disaster medicine and preparedness among health professionals have focused on TS and knowledge [10]. Therefore, it is of interest to study what is known about the use of NTS in disaster responders.

With the background of this lack of universal agreement and knowledge of which NTS are needed for the medical disaster response, this scoping review aimed to identify the NTS required for disaster responders.

## Methods

### Design

A scoping review following Arksey and O’Malley’s framework [11, 12]. 

### Data collection procedures

Arksey and O’Malley describe the methodological framework in six steps, the last of which is optional: (1) specify the research question, (2) identify relevant literature, (3) select studies, (4) chart the data, (5) summarize and report the result and (6) include expert consultation [11].

1. Research question: Which non-technical skills are required for disaster responders?
2. Relevant literature: The research team discussed and developed the search strategy in collaboration with academic librarians. Structured searches were conducted in the PubMed, Web of science, CINAHL Full Plus, PsycInfo, and Scopus databases. Pilot searches using MeSH terms were conducted first but were found to retrieve too many papers (about 300 000). Therefore, structured searches based on keywords or index words were used. (see Appendix 1).
3. Study selection: Studies were included if they described or discussed NTS needed or used by medical disaster responders, including all healthcare professions, in any disaster context. Studies with qualitative, quantitative, or case study design published in English during the years 2012–2022 were included. Study protocols or reviews were excluded. The Covidence program was used to select studies and extract data. All papers identified in the literature search were exported to the Covidence systematic review software, where duplicates were sought and removed. Thereafter, two authors (AW and KH) screened the studies, and additional studies were removed if the focus did not relate to the research question, if the study design was not qualitative, quantitative, or a case study, or if a study was not written in English. The remaining studies were included in the full analysis after further condensation.
4. Charting the data: A data extraction form was created using the Numbers program which allowed us to summarize the data in several ways. Extraction was done by one author (AW), confirmed by a second author (KH) and contextualized by a third author (LK). The information recorded included the authors, country, study design, year, event, study population, numbers of participants, and outcomes. (see Table 1).
5. Collating, summarizing and reporting the results: First, a numerical analysis of the NTS was performed. The results were then synthesized and reported according to themes that described the four most frequently mentioned NTS.

### Ethical considerations

Ethical approval was not applicable, as the study was a scoping review.

## Results

**Fig. 1:**
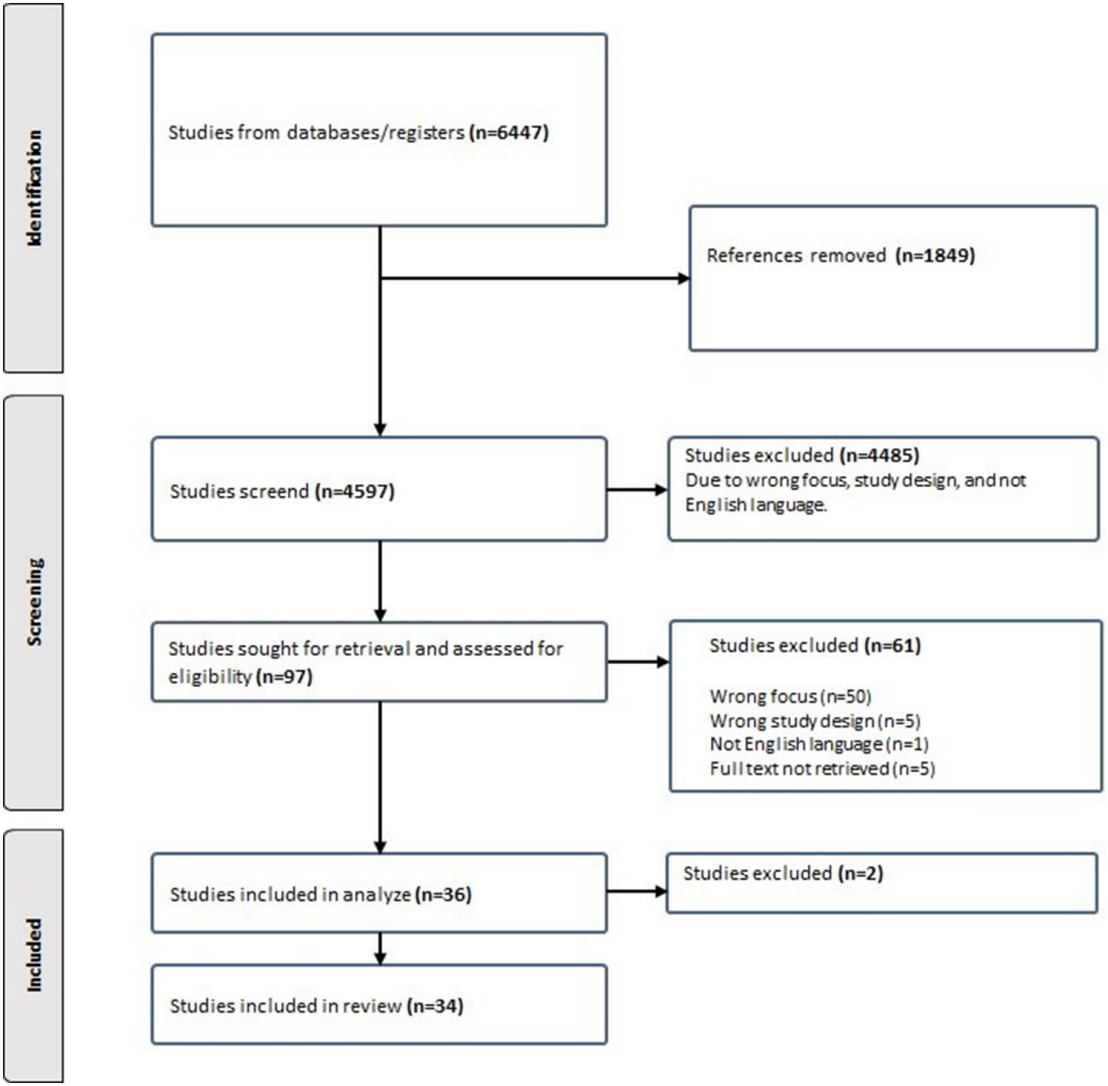
Flowchart of search result

The initial search resulted in 6447 articles. First, 1849 doubles were removed. Thereafter, 4597 studies were screened, and an additional 4485 were removed due to wrong focus for the research question, an irrelevant study design, or not being written in English. Further condensation resulted in the 34 studies included in the study. (see Fig. 1).

The most common study design was qualitative (*n* = 15), followed by cross-sectional studies (*n* = 12). The countries where the studies were conducted ranged from Australia to India and Iran. (see Table 1). In all, 15 of the 34 studies were based on actual disaster events, and the most frequent type of disaster was an earthquake. The context for the remaining 19 studies varied from training or a more general prior experience among the study participants. In seven studies the participants were solely nurses [13–19], and in two studies, only physicians were included [20, 21] One study included only pharmacists [22], and one included only students [23]. In nine studies, physicians, nurses and/or pre-hospital personnel participated [24–31], while other studies did not specify the study participants’s occupation. A total of 2087 study persons were involved in the current review (mean 77 study participants per study). However, several studies (*n* = 7) did not specify the number of the participants. Uniform use of terms such as skills or competence in the analyzed articles was lacking. None of the articles used validated tools to evaluate the various NTS.

### Most common NTS reported

**Fig. 2:**
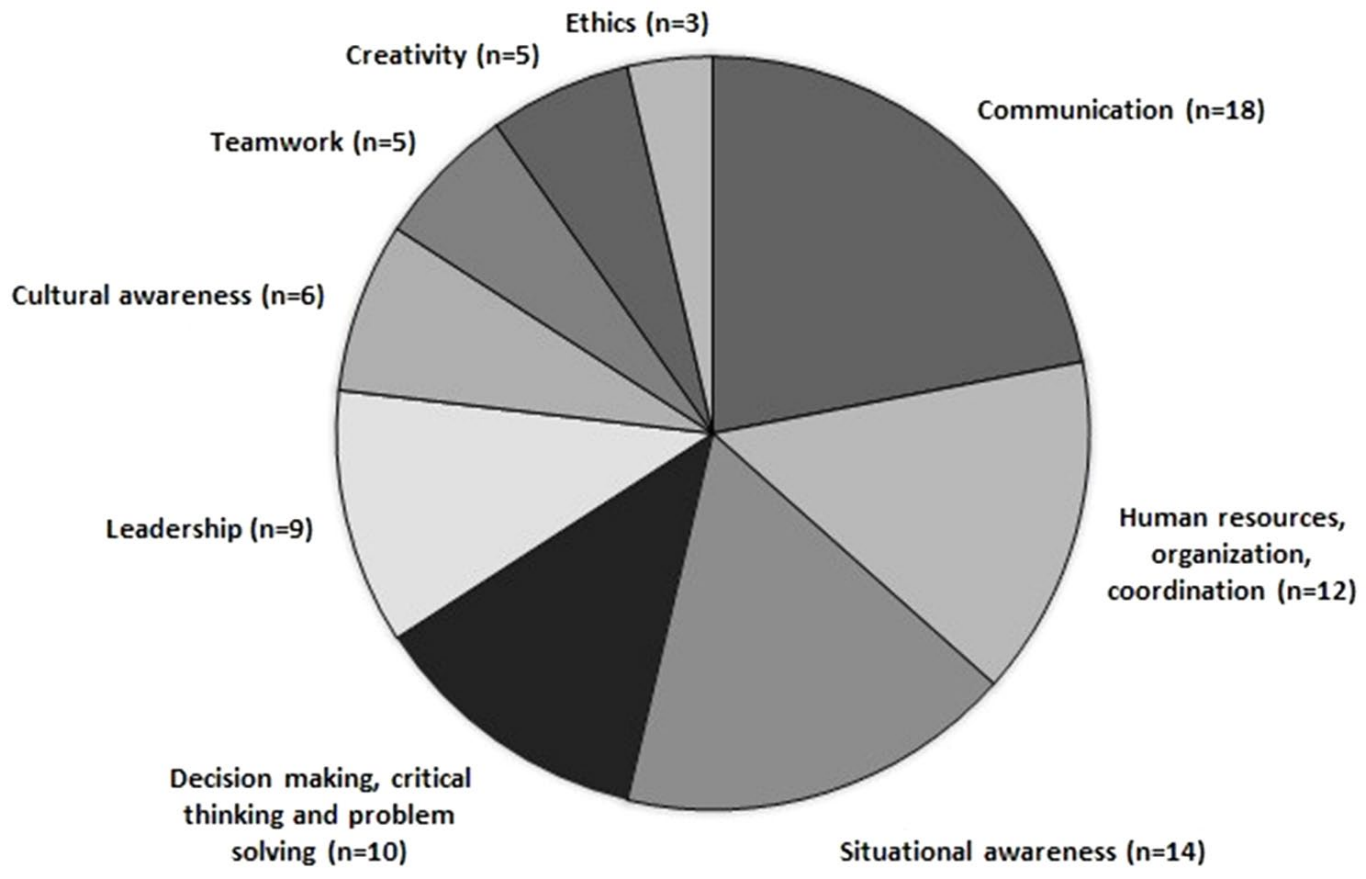
Overview of the numbers of times NTS were reported in the reviewed articles

Nine separate skills could be identified in the articles: communication skills; situational awareness; knowledge of human resources (HR) and organization and coordination skills; decision-making, critical-thinking and problem-solving skills; leadership; cultural awareness; teamwork; creativity skills; and ethics. (see Fig. 2). The four most common skills expressed as needed among disaster responders were communication, situational awareness, knowledge of HR, organization and coordination, and decision-making. These will be described in the following paragraphs.

### Communication skills

Communication skills were the most frequently mentioned NTS, appearing in 18 of the 34 articles. Although communication was defined differently in the articles reviewed, communication competence and/or skills were considered important for all disaster responders regardless of their role. Several articles described communication as verbal or written one-way information, as a discussion (two-way information) or described communication skills as the ability to handle technical information [32, 33]. Others included the ability of cross-cultural understanding, the ability to show care, active listening, and language skills [34]. Communication skills were seen as an important quality internally within the organization or the team, where good communication could increase team members’ feelings of being safe and comfortable despite a severe situation. With external communication the disaster responders had first-hand contact with the affected population [16, 35] Communication was also seen as a key component in relation to reporting to higher command and other stakeholders, where a good relationship and adequate information sharing was crucial for successful relations and cooperation [27, 29, 36, 37]. The level of communication skills was found to be higher in nurses with 1–5 years of clinical experience compared to those with more than 10 years of clinical experience. The level was also greater in groups with higher academic education [19]. Communication skills were considered important NTS for all team members [24, 25, 38, 39]. 

### Situational awareness

The second most common reported NTS was the ability to create situational awareness where scene coordination and scene safety were especially crucial for the disaster response [40]. The need for an updated and informed overall awareness of the situation was reported as important for the entire disaster response team [31, 32] The individual disaster responder needed to be able to analyze the received data and recognize limitations and possibilities of the given situation, including safety, in order to create situational awareness [24, 33, 36, 39]. Some studies suggested that this competence was most important for the leader, as leaders depended on this skill to both report to higher management levels and to adapt the response to the situation. Situation awareness also included understanding both actual and potential health hazards before, during, and after a disaster [29]. 

#### Knowledge of human resources, organization, and coordination skills

The ability to assess needs and coordinate and plan the medical response accordingly required the coordination and organization of medical teams in relation to available HR [35, 40]. In addition, the coordination skills covered coordination of other sources (e.g. supplies and equipment) and the knowledge of how to distribute such items in disasters [33]. How the team was constructed were also important, as were the skills the individual disaster responders possessed and contributed to team performance [30]. HR management was important for the ability to maintain control of the situation, to understand and value the team members, to empower the staff, and to be receptive to input and feedback. However, these skills were not always present among the disaster responders [36, 38]. Understanding the ability and the situation of local medical teams and how international response teams could support and integrate with local colleagues was stressed, as this required substantial coordination and organization skills [28]. 

### Decision-making, critical thinking and problem-solving skills

Decision-making skills include both individual and joint decision-making within a team. Problem-solving and decision-making were the most essential attributes for a team leader but only in sixth (out of ten) place for team members [25]. Decision-making was pointed out as often depending on other partners and therefor complicated [32]. The ability to make decisions and solve problems was important from the team safety perspective and depended on correct situational awareness [39]. 

## Discussion

This scoping review of 34 studies identified four NTS that were most frequently mentioned as important for disaster responders: communication skills; situational awareness; knowledge of HR, organization, and coordination skills; and decision-making, critical thinking, and problem-solving skills.

In line with previous research, *communication skills* were found to be the most mentioned NTS in the current review [41]. Communication skills provide a foundation for other NTS, such as leadership and team coordination. Communication is a broad topic which refers to both verbally and written interaction, and in addition to that it also contains the individual’s ability as well as general knowledge of communication systems. Similarly, *situational awareness* is essential for both team members and leader [42, 43]. Although *knowledge of HR, organization, and coordination skills* were highly desired, this was also the NTS most often reported as lacking. *Decision-making, critical thinking, and problem-solving* are other qualities that are important mainly for a team leader. However, in order to understand why decisions and adherence to them were important, it was also crucial for team members to understand this process. These findings confirms that NTS are essential for disaster responders.

The terms used to describe NTS varied widely in the included studies. This inconsistent use of terms and lack of agreement on important components of disaster medicine competence have been raised before and are again confirmed by this scoping review [3]. The lack of uniform use of terms like skills or competence has presented challenges to synthesizing the studies used in this review. This made it harder to draw conclusions when analyzing the articles. Several studies used a moderately small study sample, and a number of articles did not clearly state what scientific method had been used, while other articles held a high scientific level. In 19 of the 34 articles, the results relied on a training setting rather than on an actual event, which raises concerns about the applicability of identified skills to actual events.

The studies included in the review do not give a homogenous result on whether NTS are important for the individual or the team; however, previous studies within the health care sphere have concluded that NTS are important for both the team and team performance [44, 45]. The Katz management model, later modified by Mumford, suggests the close relationship of TS, NTS and higher understanding of the task as vital parts for both team members and the team leader [46, 47]. Though focused on leadership qualities, the model involves the leader and followers and could be applied in the disaster medicine setting. Together with frameworks already developed, this model could be used to develop a framework for disaster responders and disaster response teams regarding NTS.

Despite the literature on the impact and necessity of NTS in the health care system indicating that they are non-negotiable, NTS are still not being fully prioritized [5, 48, 49]. This review emphasizes the need for NTS to be included as an essential part of disaster medicine competence. For example, NTS should be considered an important part of the training of disaster responders, such as Emergency Medical Teams (EMTs) [50]. Consensus on how to measure and train on non-technical skills is needed. Evaluation tools for NTS have been developed for some occupations, for example HeliNOTS for helicopter pilots, ANTS for anesthetics, and NOTSS for surgeons, mainly focusing on simulated situations [51–53]. The development and refinement of NTS have, in some professional areas, led to frameworks with skill taxonomies and behavior rating systems, which are used to find and promote these skills and behaviors [54]. However, such tools do not exists for disaster responders or in disaster medicine training.

The results of this review strongly suggest that NTS are important factors and more research focused on disaster responders and NTS is needed. It is likely that disaster medicine would benefit from frameworks and common definitions, both for disaster responders at work and in disaster medicine training. In addition, future research should rely on scientifically rigorous methods, strive to use larger study samples, and be based on real disaster events rather than trainings.

### Limitations

The lack of indexed search terms required a somewhat untraditional search strategy, for example without including MeSH terms, to find papers to include in this review. The term “non-technical skills” could have been used, but since it was found to render fewer than 50 papers, the broader term “competence” was used instead. However, given the lack of indexed keywords, more than 4500 papers from several databases were screened. Even so, it cannot be ruled out that more papers might have been identified using another search strategy. The heterogeneity among the included studies also complicated the analysis and alternative ways to analyze the results cannot be excluded. To validate the analysis, a research team, comprising individuals with both theoretical and practical experiences from disaster response was used.

## Conclusions

NTS are crucial for disaster responders. The most studied NTS were communication skills; situational awareness; knowledge of HR and organization and coordination skills; decision-making, critical-thinking and problem-solving skills. NTS has proven to be trainable skills in other settings, and it is likely that disaster responders training would benefit from training of NTS. To increase knowledge on how to incorporate NTS in recruitment processes and disaster medicine trainings, future research should rely on scientifically rigorous methods, strive to use larger study samples and be based on real disaster events rather than training settings.

## Appendix 1. Search strategy

### Pubmed (NLM)

Search performed November 15, 2022.

**Table:** 

	1.	Search: ((disasters[MeSH Terms]) OR (disaster*[Title/Abstract])) AND (“skills“[Title/Abstract]) Filters: English, from 2012 to 2022

### Web of Science

Search performed November 16, 2022.

Indexes = SCI-EXPANDED, SSCI, A&HCI, CPCI-S, CPCI-SSH, ESCI Timespan = All years.

**Table:** 

	1.	(ALL=(Disaster*)) AND ALL=(Skills) OR Mass casualty*=(competencies)
	2.	(ALL=(Disaster*)) AND ALL=(Skills)

### Scopus

(Elsevier). Search performed November 16, 2022.

**Table:** 

	1.	(TITLE-ABS-KEY (Disaster*) AND TITLE-ABS-KEY (Mass casualty*) AND TITLE-ABS-KEY (competencies )
	2.	TITLE-ABS-KEY (Disaster*) AND TITLE-ABS-KEY (Skills)
Limits		
	8.	PUBYEAR > 2012 AND PUBYEAR < 2022, English, Article, Review

### CINAHL fulltext

Search performed November 16, 2022.

**Table:** 

	1.	((disaster*) AND (mass casualty*) AND (competencies))
	2.	((disaster*) AND (skills))
Limits		
	3.	(Publication Years 2012–2022). Exclude pre-cinahl, medline records

### PsychInfo

Search performed November 16, 2022.

**Table:** 

	1.	((disaster*) OR (mass casualty*) AND (competence*)) OR (skills*))
Limits		
	3.	(Publication Years 2012–2022).

### Electronic supplementary material

Below is the link to the electronic supplementary material.

Supplementary Material 1

Supplementary Material 2

## Data Availability

Not applicable.

## Abbreviations

HR: Human resources
EMT: Emergency Medical Team
NTS: Non-technical skills
TS: technical skills
