# Supplementary material for: Non-technical skills needed by medical disaster responders– a scoping review
Source: Scand J Trauma Resusc Emerg Med. 2024 Apr 2;32:25. doi: 10.1186/s13049-024-01197-y (PMC10988843; doi:10.1186/s13049-024-01197-y)
Supplement: Supplementary file 1 — Supplementary Material 1 [file 13049_2024_1197_MOESM1_ESM.docx]

**Table 1**

*34 studies included in the scoping review.*

| *Authors and year of publication* | *Country* | *Study design* | *Event* | *Study population* | *No of participants* | *NTS reported as important* |
| --- | --- | --- | --- | --- | --- | --- |
| Abbasi et al  2003(1) | Iran | Case studies | Earthquake | Physician, Nurse, Undefined Disaster responder | Not stated | Not stated |
| Akbari et al  2018(2) | Iran | Qualitative | Not specified | Nurse | 35 | HR, organization and coordination  Situational awareness |
| Azizpour et al  2022(3) | Iran | Cross sectional | No event | Nurse, pre-hospital personnel | 472 | Decision-making, critical thinking, and problem-solving |
| Black et al  2022(4) | UK? | Case reposrt | Multiple | Physician, Undefined Disaster responder | 10 | Comunication  Situational awareness  Decision-making, critical thinking, and problem-solving |
| Brooks et al  2019(5) | Australia | Case studies | Not specified | Not specified | Not stated | Divergent thinking |
| Chegini et al  2022(6) | Iran | Qualitative | No event | Nurse | 271 | Communication  Decision-making, critical thinking, and problem-solving |
| Cole et al  2020(7) | UK | Qualitative | Various | Undefined Disaster responder | 8 | Cultural awareness |
| Cox et al  2014(8) | USA | Special report | No event | Nurse | Not stated | Leadership |
| Deitchman et al  2013(9) | Philippines | Cross sectional | No event | Undefined Disaster responder | 272 | Communication  Situational awareness |
| Goniewicz et al  2021(10) | Poland | Cross sectional | No event | Physician, Nurse, Pre-hospital | 134 | Not stated. |
| Goodwin et al  2017(11) | USA | Mixed methods | No event | Nurse, Undefined Disaster responder | 40 | Communication |
| Hu et al  2020(12) | China | Qualitative | No event | Undefined Disaster responder | 20 | Communication  Situational Awareness  HR, organization and coordination  Cultural awareness  Leadership |
| Kiani et al  2017(13) | Iran | Qualitative | Previous experience | Undefined Disaster responder | 21 | Interpersonal factors |
| King et al  2019(14) | USA | Case report | Various | Physician, Nurse, Pre-hospital, Undefined Disaster responder | 220 | Communication  Decision-making, critical thinking, and problem-solving  Teamwork |
| Meduri et al  2021(15) | India | Qualitative | Various | Undefined Disaster responder | 44/23 | Communication  Decision-making, critical thinking and problem-solving  Leadership |
| Nasiri et al  2019(16) | Iran | Qualitative | Various | Physician, Undefined Disaster responder | 30 | Communication  Situational awareness  HR, organization and coordination |
| Nekooei et al  2014(17) | Iran | Qualitative | Earthquake | Nurse | 23 | Not stated |
| Noguchi et al  2016(18) | Japan | Cross sectional | Various | Physician | 52 | HR, organization and coordination |
| Parekh et al  2021(19) | USA | Case study | Pandemic | Physician | Not stated | Communication  Leadership  Situational awareness  HR, organization and coordination |
| Peller et al  2013(20) | Canada | Qualitative | Various | Undefined Disaster responder | 10 | Communication  Situational Awareness  Decision-making, critical thinking and problem-solving  Teamwork  Creativity  Cultural awareness |
| Remington et al  2017(21) | USA (Haiti) | Cross sectional | Earthquake | Undefined Disaster responder | 59 | Communication  Leadership |
| Rezaei et al  2020(22) | Iran | Qualitative | Earthquake | Nurse | 16 | Communication  Creativity  Ethics |
| Rivkind et al  2015(23) | Israel | Cross sectional | Training/  No event | Student | Not stated | Not stated |
| Schultz et al  2012(24) | USA | Cross sectional | No event | Physician, Nurse, Pre-hospital, Undefined Disaster responder | 23 | Communication  Situational Awareness  HR, organization and coordination  Decision-making, critical thinking and problem-solving  Ethics |
| Skryabina et al  2021(25) | UK | Cross sectional | Terrorist attack | Physician, Nurse, Pre-hospital, Undefined Disaster responder | 21 (interview) 86 (survey) | Communication  HR, organization and coordination  Decision-making, critical thinking and problem-solving  Leadership |
| Son et al  2020(26) | USA | Qualitative | Hurricane | Undefined Disaster responder | 10 | Communication  Decision-making, critical thinking and problem-solving  Situational Awareness  Creativity |
| Vaishya et al  2015(27) | India (Nepal) | Case report | Earthquake | Physician, Nurse | Not stated | HR, organization and coordination |
| Vardanyan et al  2018(28) | Brazil | Cross sectional | Various | Pharmacist | 6 | Decision-making, critical thinking and problem-solving  Cultural awareness |
| Von Strauss et al  2017(29) | Sweden (Sierra Leone) | Cross sectional | Ebola | Nurse | 44 | Communication  Leadership  Teamwork |
| Walsh et al  2012(30) | USA | Cross sectional | No event | Physician, Pre-hospital, Undefined Disaster responder | 60 | Communication  Situational Awareness  HR, organization and coordination  Ethical |

**References**

1. Abbasi M, Salehnia MH. Disaster medical assistance teams after earthquakes in iran: propose a localized model. Iran Red Crescent Med J. 2013;15(9):829-35.

2. Akbari FA, Bahrami M, Aein F, Khankeh H. Iranian Nurses' Experience of Management Competences in Disaster Response: A Qualitative Study. PAKISTAN JOURNAL OF MEDICAL & HEALTH SCIENCES. 2018;12(4):1799-803.

3. Azizpour I, Mehri S, Soola AH. Disaster preparedness knowledge and its relationship with triage decision-making among hospital and pre-hospital emergency nurses - Ardabil, Iran. BMC Health Services Research. 2022;22(1).

4. Black A, Brown O, Utunen H, Gamhewage G, Gore J. Insights on Public Health Professionals Non-technical Skills in an Emergency Response (Multi-Team System) Environment. Frontiers in Psychology. 2022;13.

5. Brooks B, Curnin S, Owen C, Boldeman J. New human capabilities in emergency and crisis management: from non-technical skills to creativity. AUSTRALIAN JOURNAL OF EMERGENCY MANAGEMENT. 2019;34(4):23-30.

6. Chegini Z, Arab-Zozani M, Kakemam E, Lotfi M, Nobakht A, Aziz Karkan H. Disaster preparedness and core competencies among emergency nurses: A cross-sectional study. Nursing Open. 2022;9(2):1294-302.

7. Cole MJR, Barrett RV, Mein GK. UK rehabilitation professionals' experiences undertaking short-term responses after sudden-onset disaster. International Journal of Therapy & Rehabilitation. 2020;27(6):1-13.

8. Cox RS, Danford T. The need for a systematic approach to disaster psychosocial response: A suggested competency framework. Prehospital and Disaster Medicine. 2014;29(2):183-9.

9. Deitchman S. Enhancing crisis leadership in public health emergencies. Disaster Medicine and Public Health Preparedness. 2013;7(5):534-40.

10. Goniewicz K, Burkle FM, Khorram-Manesh A. The gap of knowledge and skill – One reason for unsuccessful management of mass casualty incidents and disasters. American Journal of Emergency Medicine. 2021;46:744-5.

11. Goodwin Veenema T, Deruggiero K, Losinski S, Barnett D. Hospital Administration and Nursing Leadership in Disasters. Nursing Administration Quarterly. 2017;41(2):151-63.

12. Hu X, Chen H, Yu M. Exploring the non-technical competencies for on-scene public health responders in chemical, biological, radiological, and nuclear emergencies: a qualitative study. Public Health (Elsevier). 2020;183:23-9.

13. Kiani M, Fadavi M, Khankeh H, Borhani F. Personal factors affecting ethical performance in healthcare workers during disasters and mass casualty incidents in Iran: a qualitative study. Medicine, Health Care and Philosophy. 2017;20(3):343-51.

14. King RV, Larkin GL, Klein KR, Fowler RL, Downs DL, North CS. Ranking the Attributes of Effective Disaster Responders and Leaders. Disaster Medicine and Public Health Preparedness. 2019;13(4):700-3.

15. Meduri Y. Personnel needs assessment in times of crisis: a focus on management of disasters. RAUSP MANAGEMENT JOURNAL. 2021;56(4):390-407.

16. Nasiri A, Aryankhesal A, Khankeh H. Leadership in limbo: Characteristics of successful incident commanders in health sector of a disaster-prone country. International Journal of Health Planning and Management. 2019;34(4):e1495-e509.

17. Nekooei Moghaddam M, Saeed S, Khanjani N, Arab M. Nurses' requirements for relief and casualty support in disasters: a qualitative study. Nurs Midwifery Stud. 2014;3(1):e9939.

18. Noguchi N, Inoue S, Shimanoe C, Shibayama K, Matsunaga H, Tanaka S, et al. What Kinds of Skills Are Necessary for Physicians Involved in International Disaster Response? Prehospital and Disaster Medicine. 2016;31(4):397-406.

19. Parekh VK, Swartz KL. Lessons for psychiatrists from the COVID pandemic: the need for expanded roles and additional competencies. International Review of Psychiatry. 2021;33(8):668-76.

20. Peller J, Schwartz B, Kitto S. Nonclinical core competencies and effects of interprofessional teamwork in disaster and emergency response training and practice: a pilot study. Disaster medicine and public health preparedness. 2013;7(4):395-402.

21. Remington CL, Ganapati NE. Recovery worker skills in post-earthquake Haiti: the disconnect between employer and employee perspectives. NATURAL HAZARDS. 2017;87(3):1673-90.

22. Rezaei SA, Abdi A, Akbari F, Moradi K. Nurses' professional competences in providing care to the injured in earthquake: A qualitative study. J Educ Health Promot. 2020;9:188.

23. Rivkind AI, Faroja M, Mintz Y, Pikarsky AJ, Zamir G, Elazary R, et al. Combating terror: A new paradigm in student trauma education. Journal of Trauma and Acute Care Surgery. 2015;78(2):415-21.

24. Schultz CH, Koenig KL, Whiteside M, Murray R. Development of national standardized all-hazard disaster core competencies for acute care physicians, nurses, and EMS professionals. Annals of Emergency Medicine. 2012;59(3):196-208.e1.

25. Skryabina E, Betts N, Reedy G, Riley P, Amlôt R. UK healthcare staff experiences and perceptions of a mass casualty terrorist incident response: a mixed-methods study. Emerg Med J. 2021;38(10):756-64.

26. Son C, Sasangohar F, Peres SC, Moon J. Muddling through troubled water: resilient performance of incident management teams during Hurricane Harvey. Ergonomics. 2020;63(6):643-59.

27. Vaishya R, Agarwal AK, Vijay V, Hussaini M, Singh H. Surgical Management of Musculoskeletal Injuries after 2015 Nepal Earthquake: Our Experience. Cureus. 2015;7(8):e306.

28. Vardanyan H, Mosegui GBG, Miranda ES. Skills and Core Competencies of Pharmacists in Humanitarian Assistance. Prehospital and Disaster Medicine. 2018;33(3):266-72.

29. von Strauss E, Paillard-Borg S, Holmgren J, Saaristo P. Global nursing in an Ebola viral haemorrhagic fever outbreak: before, during and after deployment. Global Health Action. 2017;10(1):N.PAG-N.PAG.

30. Walsh L, Subbarao I, Gebbie K, Schor KW, Lyznicki J, Strauss-Riggs K, et al. Core competencies for disaster medicine and public health. Disaster Medicine and Public Health Preparedness. 2012;6(1):44-52.
